# Supplementary material for: Neurophysiological evidence of efference copies to inner speech
Source: eLife. 2017 Dec 4;6:e28197. doi: 10.7554/eLife.28197 (PMC5714499; doi:10.7554/eLife.28197)
Supplement: Supplementary file 1. — The data were analysed with this syntax using the program IBM SPSS Statistics (v. 23). [file elife-28197-supp1.docx]

**SPSS Syntax for the Inner Speech Experiment: analysis of N1 amplitude**

DATASET ACTIVATE DataSet1.

GLM N1_Fz_Listen N1_Fz_Match N1_Fz_Mismatch N1_FCz_Listen N1_FCz_Match N1_FCz_Mismatch N1_Cz_Listen N1_Cz_Match N1_Cz_Mismatch

/WSFACTOR=electrode 3 Polynomial cond 3 Polynomial

/METHOD=SSTYPE(3)

/EMMEANS=TABLES(cond) COMPARE ADJ(LSD)

/PRINT=DESCRIPTIVE ETASQ

/CRITERIA=ALPHA(.05)

/WSDESIGN=electrode cond electrode*cond.
